# Cirrhotic livers reveal genetic changes in the MDM2-P14ARF system of cell cycle regulators

**DOI:** 10.1038/sj.bjc.6600238

**Published:** 2002-04-22

**Authors:** T Schlott, J G Scharf, C Gorzel, P Middel, H Spring

**Affiliations:** Department of Cytopathology, Georg-August-University, Robert-Koch-Str 40, D-37075 Goettingen, Germany; Division of Gastroenterology and Endocrinology, Georg-August-University, Robert-Koch-Str 40, D-37075 Goettingen, Germany; Division of Pathology, Georg-August-University, Robert-Koch-Str 40, D-37075 Goettingen, Germany; Biomedical Structure Analysis Group, German Cancer Research Center, Im Neuenheimer Feld 280, D-69120 Heidelberg, Germany

**Keywords:** liver cirrhosis, *MDM2*, *P14ARF*

## Abstract

The genesis of hepatocellular carcinoma is promoted by changes in the regulatory MDM2-P14ARF system. The incidence of such changes has to date not been analysed in non-tumourous livers showing regenerative proliferation. In the present study, 24 cirrhotic livers of alcohol-, autoimmue disorder- or HCV-caused genesis were screened for MDM2-P14ARF alterations at the level of protein, DNA and mRNA. Using confocal laser scanning microscopy, the absence of MDM2 and P14ARF expression was detected in all samples except three HCV-infected livers (four livers) which contained hepatocytes overexpressing MDM2 (P14ARF) protein. In two of the samples lacking P14ARF expression, laser microdissection and PCR demonstrated deletion of the *P14ARF* gene. The *P14ARF* gene amplified from other specimens did not carry mutations. *MDM2* splicing variants were present in tissues from alcohol- and autoimmune disorder-induced cirrhoses. Sequencing of full-size mRNA revealed a *MDM2* mis-sense mutation in an alcohol-induced cirrhosis. One sample contained regenerative nodules with genetic instability occurring at *MDM2* locus *D12S83* according to the data of automatic PCR fragment analysis. In summary, this study gives first evidence for different types of *MDM2* and *P14ARF* alterations in cirrhotic livers. We suggest that the changes impair the regulatory MDM2-P14ARF system, thus possibly favouring regenerative proliferation and transformation.

*British Journal of Cancer* (2002) **86**, 1290–1296. DOI: 10.1038/sj/bjc/6600238 www.bjcancer.com

© 2002 Cancer Research UK

## 

Hepatocellular carcinoma (HCC) is the most common primary malignancy of the liver and the most common cancer in some geographic areas (Simonetti *et al*, 1991). Epidemiologic studies have demonstrated a correlation between high prevalence of viral infection of the liver and the incidence of HCC. In western countries, hepatitis C virus (HCV) infection is detected in greater than 10% of patients with HCC and in up to 80% in Italy and Spain (Okuda, 1997). Additionally, in Japan an increase in HCC incidence has been attributed to an increase in HCV-associated HCC while the incidence of HBV-induced HCC has not changed much (Okuda, 1997).

Viral and toxin-induced mechanisms in the cirrhotic liver may activate oncogenes and inactivate tumour suppressor genes. In this context, the human oncogene *MDM2* (Fakharzadeh *et al*, 1991) is a candidate for promoting pathogenic changes in cirrhotic liver tissue. It encodes a phosphoprotein which inhibits DNA repair and apoptosis by forming a complex with tumour suppressor p53 (Momand *et al*, 1992). Additionally, MDM2 interacts with cell regulators such as the TATA-binding protein TBP and basal transcription factor TAFII250 (Leveillard and Wasylyk, 1997), thus emphasising the importance of MDM2 protein in the regulation of cell division. Nucleocytoplasmic MDM2 accumulation represents an important tumorigenic mechanism that immortalises fibroblasts (Finlay, 1993). In solid tumours, MDM2 overexpression is caused by *MDM2* gene amplification (Ladanyi *et al*, 1993). Other genetic *MDM2* abnormalities are mutations in tumours that might prolong the half-life of MDM2 (Schlott *et al*, 1997; 1999). Finally, *MDM2* splicing variants with transforming potential were detected in malignant neoplasms (Haines *et al*, 1994, Sigalas *et al*, 1996). Recently, the tumour suppressor P14ARF was shown to influence MDM2 function by binding and transporting MDM2 into the nucleolus for inactivation (Tao and Levine, 1999; Weber *et al*, 1999). Homozygous deletions of the *P14ARF* gene promote accumulation of MDM2 and ubiquitin ligase activity of MDM2 for tumour suppressor p53 (Honda and Yasuda, 1999). The deletions were detected in neoplasms such as lung carcinomas and malignant peripheral nerve sheath tumours and were considered to be involved in the process of tumorigenesis (Kourea *et al*, 1999; Sanchez-Cespedes *et al*, 1999).

HCC usually develops in a liver with advanced chronic liver disease and rarely develops in a normal liver. Since alterations of the MDM2/P14ARF system have been observed in many human tumours – including hepatocellular carcinoma – they may also be present in cirrhotic liver thus representing early events in the pathogenesis of the liver. The present study investigates this possibility in detail.

## MATERIALS AND METHODS

### Liver samples

Peripheral blood and fresh/paraffin-embedded biopsates from 24 cases with cirrhotic liver were collected. The protocol was approved by the ethical commitee of the University of Goettingen. All studied patients required liver transplantation because of underlying liver cirrhosis. Fresh biopsates from a case with hepatocellular carcinoma and from normal liver tissue served as control in the immunofluorescence staining. Diagnosis of cirrhosis and HCC was based on histological findings. The aetiology of liver cirrhosis was recognised as HCV-induced (*n*=10; 5 male, 5 female; HBs antigen and HBV-DNA negative), alcohol-induced (*n*=9; 7 male, 2 female; HCV and HBV negative) and autoimmune liver disease (*n*=5; 1 male, 4 female; primary biliary cirrhosis *n*=3, primary sclerosing cholangitis *n*=1, autoimmune hepatitis *n*=1; HCV and HBV negative). Neither dysplastic nodules nor hepatocellular carcinoma cells were observed in the cirrhotic tissue samples analysed.

### Immunofluorescence analysis of MDM2 and P14ARF protein expression

Single fluorescence labelling was performed with cryosections. Sections were rinsed in cold aceton (100%, −20°C) for 5 min and washed three times in PBS (pH 7.2). The coverslips were placed onto parafilm into wet chamber and blocked by incubation with human serum (DAKO, Denmark) for 30 min. Primary antibodies MDM2 (NCL-MDM2, mouse monoclonal, Novocastra, UK), P14ARF (FL-132, rabbit polyclonal, Santa Cruz Biotechnology, USA) were diluted 1 : 20 in PBS and applied onto the coverslips. After overnight incubation, the sections were washed three times in PBS on a lab shaker. Afterwards secondary antibodies were diluted in PBS 1 : 10 and added to the cryosections. MDM2 antibody was detected using anti-mouse-labelled rabbit antibody with fluorescent dye FITC, P14ARF antibody was detected with anti-rabbit-labelled swine antibody with fluorescence dye FITC (F205, DAKO, Denmark). Sections were incubated for 2 h and washed three times in PBS. For mounting of the cover slips, DAKO-R fluorescent mounting medium was used. Microscopy was performed with a Zeiss LSM 510 UV confocal microscope (Carl Zeiss, Jena, Germany). Images were taken simultaneously in transmitted light with differential interference contrast (DIC) and in the confocal fluorescence mode of the instrument. Sections from hepatocellular carcinoma/normal liver tissue served as positive/negative control.

### DNA analysis

#### DNA extraction from white blood cells

The *DNA isolation kit for mammalian blood* (Roche, Germany) was used to isolate white blood cells from peripheral blood and to extract the DNA. The protocol was performed according to the manufacturer's instructions.

#### Laser microdissection and DNA extraction

For single-cell DNA PCR, PEN membrane (PALM, Germany) was attached to glass slides by dipping the slide into 70% ethanol and pricking up a pre-cut piece of membrane. The membrane was fixed to the slide by tape. The slides were incubated with poly-L-lysine (Sigma, Munich, Germany) and dried for 1 h at 37°C. 5 μm sections were cut from formalin-fixed, paraffin-embedded sections or cryosections and transfered to the membranes. Paraffin-embedded sections were incubated in xylene for 2×15 min and rehydrated in ethanol (99%) for 2×10 min, in ethanol (96%) for 2×10 min and in ethanol (70%) for 2×10 min. All sections were counterstained with methylene blue. Regenerative nodules were isolated by laser microdissection (laser microdissection system, PALM, Bernried, Germany) according to the method of Schuetze and Lahr (1999). Cells were transferred into 10 μl of a solution consisting of master mix 1 and master mix 2 of the Expand™ High Fidelity PCR system (Roche, Mannheim, Germany) but lacking PCR enzyme mix. This solution included 4 mg ml^−1^ proteinase K (DAKO, Denmark) and 0.5% Tween 20 (Merck, Darmstadt, Germany). Cell lysis was performed by incubation at 54°C overnight. On the following day proteinase K was inactivated by heating the solution for 10 min at 94°C. To increase the quantity of whole genomic DNA, 40 μl of the Expand™ High Fidelity PCR solution were added, consisting of master mix 1, master mix 2, 5U Taq Expand High Fidelity polymerase (Roche, Mannheim, Germany), 25 mM MgCl_2_ and 5 μl 25 pmol μl^−1^ random primer 5′-CCGACTCGAGNNNNNNATGTGG-3′. In each run, test tubes were heated to 94°C for 2 min, followed by 10 PCR cycles (94°C for 15 s, 40°C for 30 s, 68°C for 4 min) and 20 PCR cycles (94°C for 15 s, 40°C for 30 s, 68°C for 4 min; cycle elongation for 5 s each cycle) and a final extension at 72°C for 7 min. The resulting amplification products were used for conventional *P14ARF* PCR and microsatellite PCR.

#### Analysis of P14ARF gene deletion and gene mutation

*P14ARF* and *MDM2* gene were co-amplified by multiplex PCR. *P14ARF* primers yielding a 174 bp product were described by Newcomb *et al* (1999). *MDM2* DNA primers were 5′-CTCAACACAAGCTGAAGAGG-3′ (sense primer) and 5′-ATTGGTTGTCTACATA CTGG-3′ (antisense primer) yielding a 399 bp product. In case of negative *P14ARF* PCR, data were reaffirmed by seminested PCR: 3 μl of the first-run PCR mixture were transfered to a reaction cup and amplified using first-run *P14ARF* sense primer (Newcomb *et al*, 1999) and primer 5′-TCACCAAGAACCTGCGCACC-3′ (novel *P14ARF* antisense primer). These primers yield a 122 bp *P14ARF* fragment. The first-run PCR solution consisted of 5 μl 10× PCR buffer (Pharmacia, Freiburg, Germany), 30 pg primer, 10 μM of each dNTP (Pharmacia, Freiburg, Germany), 1 μl aliquot of pre-amplified DNA and 1 unit of Taq DNA polymerase (Pharmacia, Freiburg, Germany). When perfoming second-run PCR, 3 μl first-run product were used instead of pre-amplified DNA. The final volume was 50 μl. Thermal cycling was performed according to Newcomb *et al* (1999). Resulting P14ARF fragments were isolated from the gel using QIA Quick PCR Purification Kit (QIAGEN, Hilden, Germany) and automatically sequenced.

#### Automatic analysis of MDM2 microsatellite instability

Microsatellite marker *D12S80* and *D12S83* flanking the *MDM2* gene were amplified using 1 μl aliquot of preamplified DNA in a final volume of 20 μl for 30 cycles. After an initial 95°C denaturation step for 1 min (3 min), cycles were performed at 94°C (1 min), 56°C (1 min), and 72°C (1 min), followed by a final extension step at 72°C for 7 min. PCR products were separated on a 3% (w v^−1^) agarose gel and stained with ethidium-bromide. The reaction mixture contained 20 pmol of each primer, 1 μl dNTP (10 mM each), 5 μl PCR buffer (10×), and 1 U Taq polymerase (Pharmacia, Germany). The primer sequences were obtained from Genethon (Paris). Sense primer was labelled with fluorescent dye HEX. For fluorescence-based analysis of fragments, 5 μl of each PCR mix were loaded onto the ABI 310 DNA Analyser (Perkin-Elmer, Germany). Additionally, internal size standard GENESCAN™, TAMRA (Perkin-Elmer, Germany) was added to the mixture. After the run, peak shifts were detected with GENESCAN software. If band shifts indicated instability, at least one repetition of PCR was performed on the same DNA sample. Criterium for loss of heterozygosity was a 50% lower ratio in signal intensity in one of the alleles when comparing tumour with matched normal sample.

### RNA analysis

#### RNA preparation

Total RNA was extracted according to Scharf *et al* (2000) and stored at −70°C.

#### MDM2 RT–PCR for detection of splicing variants

A GeneAmp RNA-PCR Kit was used according to the manufacturer's instructions (Perkin Elmer, Weiterstadt, Germany) using 1 μl RNA solution and random hexamers. For amplification of *MDM2* sequences, PCR primers were used as previously described yielding a 1536 bp product of full-size *MDM2* mRNA (Sigalas *et al*, 1996). Resulting PCR products were separated on 3% agarose gels and stained with ethidium bromide.

#### Sequencing of RT–PCR fragments

Full-size PCR fragments and fragments with deleted exons were purified using QIA Quick PCR Purification Kit (QIAGEN, Hilden, Germany). Fragments were labelled with the PRISM-Ready Reaction Dye Deoxy-TM Terminator Cycle Sequencing Kit (Applied Biosystems, Weiterstadt, Germany) and analysed in an ABI 310 analyzer.

## RESULTS

An overview of the genetic and immunohistochemical data described below is presented in Table 1

**Table 1:**
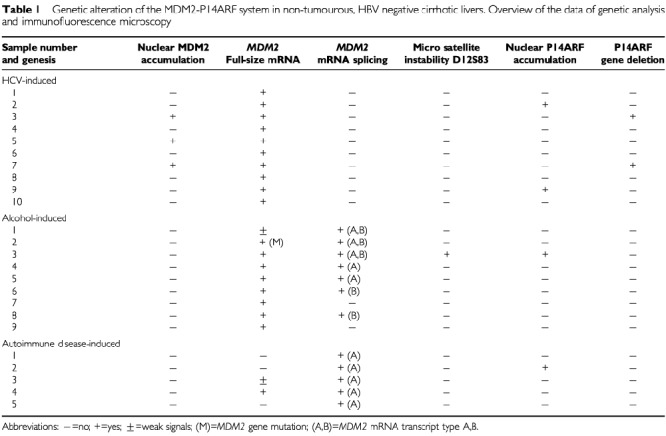
Genetic alteration of the MDM2-P14ARF system in non-tumourous, HBV negative cirrhotic livers. Overview of the data of genetic analysis and immunofluorescence microscopy

.

### MDM2 and P14ARF protein expression

**Figure 1:**
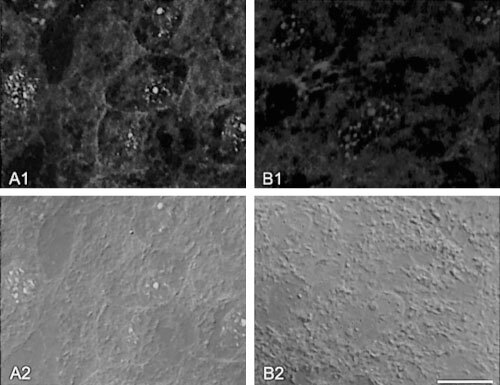
Immunofluorescence analysis of MDM2 and P14ARF expression in two cases of liver cirrhosis using confocal laser scanning microscopy. Different areas from regenerative nodules are presented containing hepatocytes overexpressing the proteins (bar: 10 μm). The upper row contains the images in confocal fluorescence. In the lower row, the fluorescence was combined with the transmitted light image in differential interference to reveal the structural aspect of the section. Pictures A1 and A2 refer to a cirrhotic liver sample showing MDM2 accumulation. Note the variations in nuclear MDM2 staining throughout the section: Some nuclei reveal absence of MDM2, others show spot-like nuclear expression, while others reveal a string-like distribution in the nucleus. The nucleoli are strongly stained. Pictures B1 and B2 contain another cirrhotic liver sample. The figures show spot-like clusters of P14ARF in the nuclei of hepatocytes.

Immunofluorescence analysis was performed on the confocal laser scanning microscope to investigate expression of the MDM2-P14ARF system in 24 cirrhotic liver tissue samples. MDM2 and P14ARF was absent in most of the samples. In regenerative nodules of three HCV-infected livers, however, interspersed hepatocytes were detected revealing spot-like overepression and nuclear accumulation of MDM2 (Figure 1

, A1, A2). Especially the nucleoli were densely filled with MDM2 protein. Notably, these samples did not express the regulatory P14ARF protein. In contrast, two other HCV-infected livers, one alcohol-induced cirrhosis and one cirrhosis induced by autoimmune disease contained areas showing overexpression of P14ARF (Figure 1, B1, B2). The distribution of hepatocytes expressing MDM2 or P14ARF was patchy. We could not detect any geographical correlates with a particular zone. Other cell types such as lymphocytes, biliary epithelium, endothelial cells, or kupffer cells did not express the MDM2-P14ARF system (data not shown).

### *MDM2* mRNA splicing and mutation

Total RNA was extracted from liver samples and analysed by RT–PCR for alternative *MDM2* mRNA splicing. Two HCV-infected samples showed 680 bp fragments. Sequencing of these PCR products indicated formation of artefacts. As a result, the HCV-infected samples did not contain *MDM2* mRNA splicing variants (data not shown).

**Figure 2:**
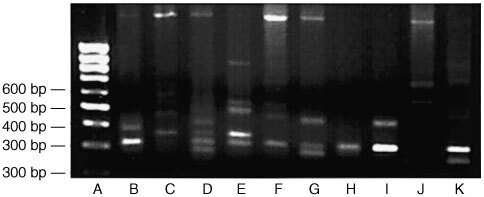
Detection of *MDM2* mRNA splicing variants in alcohol-induced cirrhoses (four samples are shown exemplarily in lane C-F; B contains positive control) and in five autoimmune disease-induced liver cirrhoses (lanes G–K). The samples were HCV negative. Lane A shows molecular weight standard. PCR fragments were separated on 3% agarose gel. The alcohol- and autoimmune disorder-induced cirrhoses express a spectrum of aberrant transcripts. Note that all tissues express full-length *MDM2* RNA of 1536 bp except samples H, I, K which only contains splicing variants of *MDM2* mRNA. Lanes A, B and E contain faint bands indicating low amounts of normal *MDM2* mRNA.

**Figure 3:**
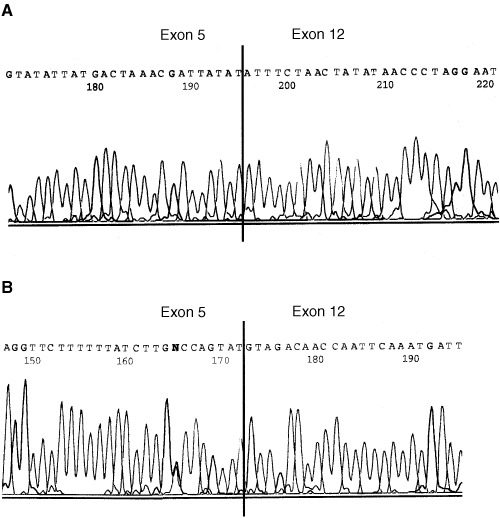
Sequence analysis of *MDM2* mRNA splicing variants performed in the automatic DNA analyser. The electropherograms indicate two RNA transcripts differing from subtype a-e published by Sigalas *et al*, 1996. mRNA (**A**) and (**B**) reveal sequence deletions between exon 5 and exon 12. Note the overlapping black and blue peak in sequence B (,,N‘‘) which may indicate heterozygous alteration (G↔C). However, reverse sequencing of this stretch resulted in normal *MDM2* sequence (data not shown).

**Figure 4:**
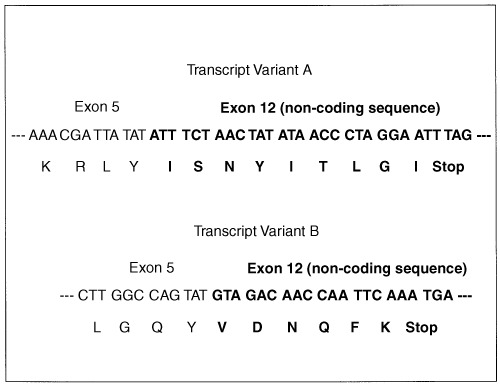
Overview on nucleotide sequence of aberrant *MDM2* mRNA splicing transcripts detected in cirrhotic livers and on the amino acid composition of the proteins coded by the transcripts. In transcript A (upper sequence), exons are deleted and the reading frame is destroyed by a non-sense mutation (stop codon). The amino acid sequence I-S-N-Y-I-T-L-G-I- is induced by a shift of the reading frame. The sequence does not occurr in wild type MDM2 protein. For concerning transcript B, bases are deleted and the reading frame is destroyed by a premature termination codon. The abnormal amino acid sequence -V-D-N-Q-F-K is the result of frame shift.

In contrast, a complex *MDM2* splicing pattern was found in 7 out of 9 alcohol- and in all autoimmune disorder-induced cirrhoses (Figure 2

). Notably, some specimens of the latter group did not contain full-size *MDM2* mRNA. Sequencing of the fragments identified two *MDM2* transcripts with deleted exons that were interspersed among amplification artefacts (Figure 3

). *MDM2* mRNA transcript A demonstrating a length of about 302 base pairs on the gel lacked bases between nucleotide position 512 (exon 5) and position 1817 (exon 12). Transcript B with a length of 387 bp lacked bases between nucleotide 491 (exon 5) and 1740 (exon 12). Transcript A was present in all positive tissues whereas transcript type B was only found in five alcohol-induced cirrhoses. The nucleotide sequences of both *MDM2* transcripts and the derived sequences of amino acids are presented in Figure 4

. The amino acid sequence showed that translation of the *MDM2* splicing variants results in proteins with premature stop codons. Additionally, the splicing caused alteration of the amino acid sequence of the last nine (transcript A) and six (transcript B) amino acid residues flanking the stop codon.

**Figure 5:**
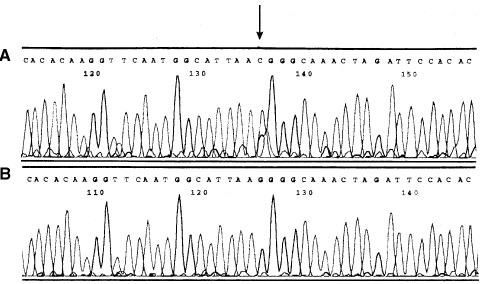
Automatic sequencing of *MDM2* mRNA isolated from an alcohol-induced cirrhosis. The upper sequence represents mutant transcript A. The sequence below contains wild type *MDM2* gene. The PCR fragments were sequenced using anti-sense primer.

A novel *MDM2* gene mutation was detected in an alcohol-induced cirrhotic liver. The mutation is located between zinc finger 1 and the C-terminal RING finger domain at position 1605 of the published sequence (Fakharzadeh *et al*, 1991). It replaces amino acid residue Leu (CTT) by Val (GTT). Interestingly, the corresponding electropherogram shows a sharp blue peak as well as a smaller black peak which represents the nucleotide of the wild type *MDM2* sequence (Figure 5

, upper sequence). It is possible that the PCR reaction contains a mixture of mutant and non-mutant MDM2 fragments. This analysis was repeated three times using different cDNA aliquots of the same liver sample to confirm the data.

### *P14ARF* gene deletion and gene mutation

**Figure 6:**
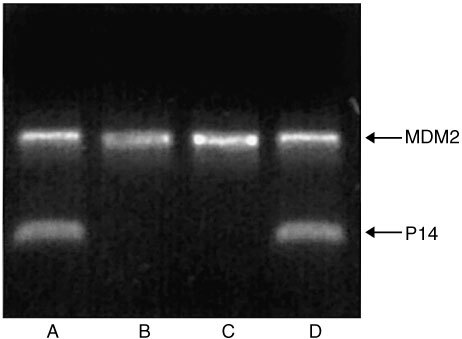
*P14ARF* gene deletion analysis in different cirrhotic livers. Hepatocytes were isolated from regenerative nodules revealing lack of P14ARF protein expression or MDM2 overexpression. DNA was isolated from microdissected cells and used for multiplex PCR. *P14ARF* sequence was coamplified with *MDM2* sequence. The upper band represents the *MDM2* fragment, the lower band shows the P14ARF fragment. In lanes A–D, 10 ng of DNA were amplified in reaction tubes. Data show that the regenerative nodules analysed in lanes B and C lack the *P14ARF* gene. These results were reaffirmed by repeating the PCR with another DNA aliquot obtained from the same nodule. Consequently, the cell cycle in these is no longer controlled by protein P14ARF.

Since most of the cirrhotic liver tissues lacked P14ARF expression, laser microdissection was performed to isolate regenerative nodules and characterise the genetic status of tumour suppressor P14ARF. Nodules were isolated from fresh and paraffin-embedded sections. *P14ARF*/*MDM2* multiplex PCR was performed and demonstrated loss of both *P14ARF* alleles in nodules isolated from two HCV-infected livers (Figure 6

). Lack of *P14ARF* gene was reaffirmed by seminested *P14ARF* PCR (data not shown). In all other samples *P14ARF* fragments were amplified which were isolated from agarose gel and automatically sequenced. Gene mutations were not detected.

### *MDM2* microsatellite instability

**Figure 7:**
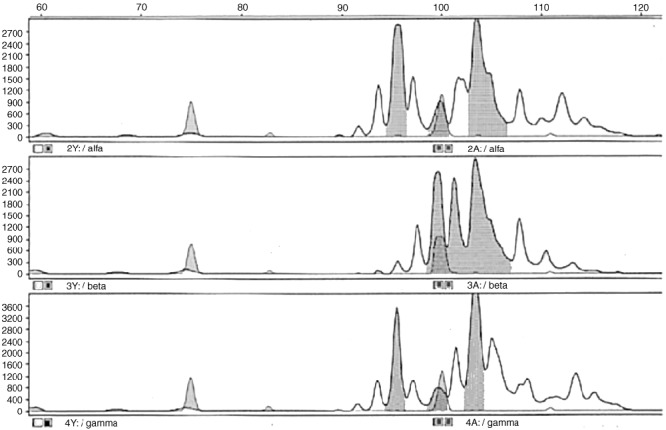
Analysis of microsatellite instability at the *MDM2* gene locus. Microsatellite marker *D12S83* was amplified using DNA isolated from normal cells and two different regenerative nodules of a cirrhotic liver sample. The fragments were amplified using sense primers labelled with fluorescence dye and sized in the ABI310 DNA analyzer by GENESCAN software. *MDM2* allele peaks and PCR fragments resulting from Taq polymerase ,,stuttering“ are presented in the electropherogram each. Upper part: electropherogram of normal human cells obtained from the patient material by microdissection. Both *MDM2* alleles are indicated by the grey peaks (95 bp and 102 bp). The red peaks refer to the internal size standard. Identical peak pattern was obtained with DNA extracted from white blood cells of the patient. Middle part: electropherogram of a regenerative nodule revealing (a) loss of the left allele peak (b) formation of two peaks of about 97 and 101 bp (c) conservation of the 102 bp allele peak. This pattern indicates chromosomal instability. Lower part: Another regenerative nodule of the same patient: The analysis shows normal pattern.

Nodules were isolated from sections of cirrhotic liver and analysed for chromosomal instability. Using PCR the microsatellite markers *D12S83* and *D12S80* were amplified which flank a stretch of about 8.2 cM, covering the entire *MDM2* gene locus at q13-14. Normal cells (white blood cells from peripheral blood, normal human cells obtained from histological sections by microdissection) served as reference. The fragments labelled with a fluorescent dye were sized using GENESCAN software of the ABI 310 Analyzer. Additionally, the quantity of each fragment was measured and compared with the fragments obtained from other regenerative nodules and from normal cells. Gain of copy number or loss of heterozygosity was not detected. However, band shifting was observed for locus *D12S83* in a regenerative nodule from a cirrhosis with alcohol-induced genesis, indicating chromosomal instability (Figure 7

).

## DISCUSSION

The pathogenesis of hepatocellular carcinoma is a focal and stepwise process involving formation of preneoplastic and neoplastic lesions in cirrhotic tissue. One of the early events in liver carcinogenesis is p53 overexpression, which has been detected in nodules of non-tumourous liver cirrhosis and which is considered to indicate a cellular stress response preceding manifestation of HCC (Livni *et al*, 1995). P53 is bound by oncoprotein MDM2 which may also contribute to hepatocarcinogenesis since MDM2 overexpression as a potential tumorigenic event was demonstrated in preneoplastic rat hepatocytes as well as in human HCC (Endo *et al*, 2000; Van Gijssel *et al*, 2000).

The present study proves that MDM2 accumulation is also a feature of some regenerative nodules in human cirrhotic livers. Since MDM2 was overexpressed exclusively in samples infected with HCV, one may speculate that viral products interacting with cellular regulators trigger MDM2 expression. The core protein of HCV, for example, was recently shown to repress transcription of regulator p21, thus promoting the proliferation of hepatocytes (Ray *et al*, 1998). We speculate that MDM2 accumulation may be a side effect of comparable protein interactions. Another explanation for the MDM2 protein overexpression is the loss of control by the *P14ARF* gene which was deleted in two out of three cases revealing MDM2 accumulation. The overexpression was not caused by *MDM2* mutations formerly found in hepatocellular carcinoma (Schlott *et al*, 1997, 1999) since none of the three samples accumulating MDM2 demonstrated corresponding changes. However, a novel *MDM2* gene mutation was found in a non-expressing, alcohol-induced cirrhosis. This result gives first evidence that benign proliferative hepatocytes in cirrhotic tissue also contain *MDM2* gene mutation. The alteration causes a Leu→Val conversion in MDM2 protein and may influence secondary structure and function of the regulatory zinc finger region.

Most of the cirrhotic livers – including the samples overexpressing MDM2 protein – appeared to lack P14ARF protein as could be concluded by the immunofluorescence staining with a single antibody. In some of the regenerative nodules, this effect was caused by homozygous *P14ARF* gene deletions. Comparable deletions were described in hepatocellular carcinoma for the *P16(INK4A)* gene encoding P14ARF protein by alternative splicing (Jin *et al*, 2000). In liver cirrhosis, the affected hepatocytes may be more susceptible to tumorigenic stimuli leading to MDM2 overepression since MDM2 can no longer be bound by P14ARF and transported into the nuclei for inactivation (Tao and Levine, 1999; Weber *et al*, 1999). Inactivating *P14ARF* gene mutations recently found in a collective of osteosarcomas and Ewing sarcomas (Tsuchiya *et al*, 2000) may be behind the lack of P14ARF expression in the other regenerative nodules. However, our data did not reveal *P14ARF* gene mutations, though destabilising mutations may still be located in other parts of the *P14ARF* gene.

To our knowledge, this is the first report on aberrant splicing of *MDM2* mRNA in liver cirrhosis. The transcript composition revealed that important motifs were deleted. For example, transcripts A and B lacked the ID1 and ID2 sequence that code for domains inhibiting cell cycle progression (Brown *et al*, 1998). It is thus possible that these proteins function as accelerators of cell division. Remarkably, in some of the samples only altered MDM2 molecules – not the normal 90 kDa MDM2 proteins – must have been present since in three cases of autoimmune disease-induced cirrhosis the aberrant *MDM2* mRNA type A was found but not the normal full-size *MDM2* mRNA. This disproportion of MDM2 proteins, to our knowledge not yet described in the literature, may influence cell regulation and respectively tumorigenesis. It is not clear why alcohol- or autoimmune disorder-induced cirrhosis but none of the HCV-induced cases showed *MDM2* mRNA splicing. This significant result may indicate that HCV infections suppress some unknown mechanisms of *MDM2* mRNA splicing. Moreover, this study provides a first clue to *MDM2* splice variant analysis in cirrhosis of different origins. The HCV-induced cases were negative and transcript B was only detected in alcohol-induced cirrhosis. Analyses of a larger collective of patients are necessary to determine whether the *MDM2* mRNA splicing pattern could be of diagnostic relevance.

Another phenomenon known to be associated with liver cirrhosis as well as hepatocellular carcinoma is microsatellite instability and LOH in the DNA repair genes *MLH1*/*MSH2* and in the tumour suppressor gene *Rb* (Ashida *et al*, 1997; Macdonald *et al*, 1998). LOH in the tumour suppressor genes *p53*, *Rb1*, *EXT1* and *APC* has been associated with hepatocellular carcinomas (Piao *et al*, 1997). In the present study, a microsatellite marker flanking the *MDM2* gene revealed instability in regenerative nodules isolated from a cirrhotic sample. It is possible that genetic instability at the *MDM2* gene locus represents another feature of hepatocytes losing control over cell cycle regulation. This finding is sufficient ground for analysing liver cirrhoses with a panel of microsatellite markers covering the entire *MDM2* gene locus to more accurately characterise chromosomal imbalance at chromosome 12q13-14.

In summary, hepatocytes in regenerative liver nodules reveal changes in the MDM2-P14ARF system that are often observed in human tumours. We speculate that the alterations may influence the function and stochiometry of both regulators, thus promoting cell proliferation and genesis of liver changes. Further analyses on a larger pool of cirrhoses may help define the diagnostic and prognostic importance of MDM2-P14ARF changes.
